# Retrospective Assessment of LLM Decision-Making for IOL Power Selection in Cataract Surgery in Comparison with Surgeon Performance

**DOI:** 10.3390/jcm15197463

**Published:** 2026-09-25

**Authors:** Randle M. Uyeda, Josh Zhe, Mina M. Sitto, Sanjana Molleti, Hanna Pawlowski, Phillip C. Hoopes, Majid Moshirfar

**Affiliations:** 1 College of Medicine—Phoenix, University of Arizona, Phoenix, AZ 85004, USA; 2 John A. Moran Eye Center, University of Utah Health, Salt Lake City, UT 84132, USA; 3 Hoopes Moshirfar Research Center, Hoopes Vision, Draper, UT 84020, USA; 4 Department of Ophthalmology and Visual Neurosciences, University of Minnesota, Minneapolis, MN 55455, USA; 5 Utah Lions Eye Bank, Murray, UT 84107, USA

**Keywords:** artificial intelligence, generative AI, GPT, toric, LLM

## Abstract

**Objectives:** To evaluate a general-purpose large language model (LLM)’s ability to perform surgeon-level decision-making in choosing an intraocular lens (IOL) power when provided the same preoperative data. **Methods:** This study included 293 eyes (174 patients) that underwent monofocal or toric cataract surgery performed by a single surgeon. GPT-5.2 analyzed the same biometric printouts, topographic displays, and preoperative data used in surgical planning. Postoperative refraction for GPT-5.2-selected IOLs was simulated from the measured postoperative refraction using a fixed conversion factor. The mean refractive deviation from target, mean absolute error (MAE), and number of eyes within ±0.25 D and ±0.50 D of target refraction were calculated. A subgroup analysis evaluated toric IOL cylinder magnitude and axis. **Results:** GPT-5.2-selected IOL power differed significantly from that of the surgeon (19.24 ± 4.01 vs. 19.64 ± 4.07 D [mean ± SD], *p* < 0.001). Using simulated refractive outcomes for GPT-5.2, MAE was higher than for surgeon-selected IOLs (0.45 ± 0.03 vs. 0.35 ± 0.02 D [mean ± SE], *p* = 0.002), and a greater percentage of eyes fell within ±0.25 D of target refraction for the surgeon (51.9% vs. 44.7%, *p* < 0.01), with no difference within ±0.50 D (*p* = 0.06). In toric eyes, the cylinder magnitude did not differ, but simulated residual astigmatism was higher for GPT-5.2 (0.78 ± 0.55 vs. 0.58 ± 0.47 D, *p* < 0.001), with an axis flip in 47.1% versus 39.2% of eyes. **Conclusions:** GPT-5.2 synthesized clinical data and approximated surgeon IOL selection, but its simulated refractive outcomes were less accurate than the surgeon’s measured outcomes. These findings suggest that a domain-specific LLM may serve as a support tool in IOL selection and cataract surgery planning.

## 1. Introduction

Cataract surgery has evolved from a vision-restorative procedure to a refractive intervention, placing greater emphasis on accurate intraocular lens (IOL) power selection [1]. Refractive outcomes depend on predicting postoperative effective lens position, which is influenced by optical biometry, anatomical parameters, and IOL constant optimization [2]. Modern formulas incorporate additional biometric measurements beyond axial length (AL), including anterior chamber depth (ACD) and lens thickness (LT), and utilize machine learning to improve IOL power accuracy [3]. Despite advances in formula design, residual refractive error persists due to limitations in biometric variability, assumptions regarding postoperative lens position, and challenges in accounting for anatomic variability [4,5].

IOL power selection is not determined solely by formula output [6,7]. Surgeons integrate formula recommendations with clinical experience, patient-specific factors, and risk tolerance, particularly when formulas diverge or biometric inputs are less reliable [6,7]. The implanted IOL, therefore, reflects an integrated clinical decision-making process.

Large language models (LLMs) are a class of general-purpose artificial intelligence (AI) systems capable of synthesizing information, performing multi-step reasoning, and generating context-aware responses [8,9,10]. Although not designed for medical applications, they require no domain-specific adaptation and have demonstrated performance on structured medical benchmarks, including clinical vignettes and board-style examinations across multiple specialties [8,9,10]. Within ophthalmology, AI has been explored in task-specific prediction models and image-based diagnostics, such as fundus photography and optical coherence tomography analysis [9,11]. Recent work has begun to apply multimodal LLMs to cataract surgical planning, including extraction of biometric values from IOLMaster printouts [12], selection of IOL type against expert consensus [13], and toric candidacy and axis planning [14]. These studies assessed data extraction, categorical IOL-type choice, or agreement with printed formula output. Whether a general-purpose LLM can synthesize this data into a final IOL power and toric selection, and how that selection performs against actual postoperative refraction, remains unclear. This study aims to compare the performance of a publicly accessible, general-purpose LLM in IOL power selection against surgeon-selected IOLs and postoperative refractive outcomes. The objective was not to evaluate its ability to perform IOL calculations, but to determine whether its recommendations align with the clinical decisions made by an experienced surgeon in routine cataract surgery, when provided with identical clinical information.

## 2. Methods

This retrospective study analyzed patients who underwent cataract extraction in one or both eyes at a single refractive surgery center in Draper, Utah. All surgeries included implantation of monofocal or toric IOLs by a single surgeon (MM) between November 2022 and November 2025. The study adhered to the tenets of the Declaration of Helsinki and received Institutional Review Board approval by the Biomedical Research Alliance of New York (#A20-12-547-823). Informed consent was obtained for the use of deidentified patient data. This study was reported in accordance with the TRIPOD-LLM reporting guideline for studies using large language models; the completed checklist is provided as Supplemental File S4.

Exclusion criteria included prior corneal refractive surgery, corneal disease, ocular comorbidities that limit refractive accuracy (e.g., glaucoma, age-related macular degeneration, or keratoconus), intraoperative complications, or implantation of IOLs other than monofocal or toric (e.g., multifocal, extended depth of focus, or light-adjustable lenses).

Preoperative images and data were collected, including sex, age at surgery, operative eye, manifest spherical equivalent (SEQ), optical biometry printouts, implanted IOL type and power, model-specific A-constants retrieved from manufacturers, and planned target SEQ. Postoperative SEQ was recorded between 3 and 30 weeks, reflecting routine clinical follow-up in a retrospective cohort. Refraction was stable within 0.5 D of its final value by approximately one week after uncomplicated cataract surgery [15]. For eyes receiving toric IOLs, postoperative cylinder and axis, IOL model designation (excluding power-specific suffixes, e.g., ZCU rather than ZCU225), and corresponding commercially available toric cylinder options were also recorded.

Optical biometry was performed using the IOL Master 700 (Carl Zeiss Meditec AG, Jena, Germany) and/or the Lenstar LS 900 (Haag-Streit, Köniz, Switzerland), with measurements from both devices reviewed when available. IOL power formula outputs (e.g., Barrett Universal II, Hill-RBF, Kane, SRK/T, Holladay 1, Haigis, EVO2.0, Hoffer Q, Olsen, and/or PEARL-DGS) were obtained from device reports. Corneal topography was performed using Pentacam HR (Oculus Surgical, Inc., Arlington, WA, USA), OPD-Scan III (Nidek Co., Ltd., Gamagori, Japan), or Galilei G4 (Ziemer USA, Inc., Alton, IL, USA).

The surgeon’s IOL power selection process involved a review of all available preoperative imaging and data. Greater weight was given to formulas demonstrating agreement, as well as the average across formulas. When targeting emmetropia, there was a preference for mild myopia to reduce the risk of hyperopic surprise. Toric IOL selection considered the degree of agreement among available toric recommendations and incorporated information from biometry and corneal topography reports.

### 2.1. GPT Prompt Design

A publicly available AI model, GPT-5.2, snapshot gpt-5.2-2025-12-11 (OpenAI, knowledge cutoff date 31 August 2025), was accessed through the application programming interface (API) between 1–30 January 2026 to generate a structured query process. A refined prompt was designed through meta-prompt engineering to optimize interactions with GPT-5.2 [16]. An initial base prompt was created and evaluated using a meta-prompt to improve the question’s structure and to generate an output that is comprehensive and aligned with the study’s objective. Preliminary prompts targeted one deficiency per revision presented to GPT-5.2 and included multiple iterations before the final execution. Prompt development was performed using a separate set of 10 eyes that met exclusion criteria and were not included in the analyzed cohort. The final, refined prompt was audited for bias and subject to defined constraints to reduce reliance on heuristic shortcuts or partial inputs.

GPT-5.2 was provided with the same retrospective dataset available to the operating surgeon at the time of surgical planning. The model was not instructed to perform calculations based on IOL formulas. GPT-5.2 was instructed to synthesize all available preoperative clinical information and generate recommendations for IOL power selection, and for toric IOLs, optimal cylinder power and axis. The structured prompt template included placeholders for patient demographics, preoperative manifest refraction, and planned IOL parameters (Supplementary File S1), which were retrieved for all eyes in the dataset. Specifically, the prompt supplied the planned IOL manufacturer and model, available toric cylinder options, and the intended refractive target. In addition to numerical data, scans from Lenstar and IOL Master 700 including formula-predicted refractions across a range of IOL powers, as well as topography reports, were uploaded (Supplementary File S2). All scans were deidentified prior to use. Each query was performed independently without using prior memory. API temperature parameters were set to 0 to reduce output variability [17]. A random subgroup of 100 eyes was selected for repeatability analysis and re-queried three times. 

A subanalysis of eyes receiving toric IOLs was performed to evaluate GPT-5.2-selected cylinder power and axis placement. Predefined assumptions were specified based on surgeon experience, including surgically induced astigmatism of 0.1 and incisions made along the horizontal meridian (180° in right eyes and 0° in left eyes).

The GPT-5.2 output included recommended spherical IOL power, toric cylinder power, and axis placement when applicable.

### 2.2. Derivation of Theoretical AI Postoperative SEQ

A 1.0 D difference in IOL power may produce 0.6–0.8 D of refractive error at the spectacle plane when accounting for factors such as axial length and effective lens position [18]. However, variation of these parameters was limited within our cohort and differences between the AI-selected and surgeon-selected IOL powers were generally small. Therefore, a fixed conversion factor of 0.7 D was used for standardized comparison between groups.

Theoretical AI postoperative SEQ = (Implanted IOL power − AI-selected IOL power) × 0.7 + postoperative SEQ.

### 2.3. Astigmatic Outcomes

Astigmatic outcomes corresponding to the surgeon- and AI-generated toric recommendations were evaluated retrospectively using the “remove and replace” method described by Hill and Potvin [19]. Preoperative refractive astigmatism, as well as surgeon-selected and AI-selected cylinder power and placement axis, were converted to vector components (J0 and J45) for analysis. The surgeon implanted IOL effect in vector form was added to the postoperative refractive (effectively removing the effect of the implanted IOL), then the AI recommended toric IOL effect was subtracted from this sum (implanting the new theoretical IOL). This yields the theoretical postoperative refraction if the AI selected IOL was implanted. Differences between AI-selected (theoretical) and surgeon-selected toric IOL outcomes were then plotted on double-angle plots to assess the magnitude and directional distribution of residual astigmatism.

Axis flip was evaluated as a categorical outcome and defined as a difference of >45° between the preoperative and postoperative astigmatism axes. The frequencies of axis preservation and axis flip were compared between GPT-5.2-generated and surgeon-selected IOLs.

### 2.4. Statistical Analysis

Statistical analyses were performed using Microsoft Excel (v16.106; Microsoft Corp., Redmond, WA, USA) and Python ver 3.11.7 (Statsmodels ver 0.14.6 and Matplotlib 3.10.8). To account for inter-eye correlation, generalized estimating equations (GEEs) with an exchangeable working correlation structure and robust standard errors were used. Each eye contributed two observations, one per selection method (surgeon and GPT-5.2), with method entered as a within-cluster factor. Clustering was specified at the patient level, so that both selection methods and both eyes of bilateral cases fell within the same cluster, accounting for the paired within-eye structure as well as inter-eye correlation. Descriptive data are presented as mean ± SD. Model-based estimates from GEE are presented as estimate ± robust standard error. A *p*-value < 0.05 was considered statistically significant.

Mean prediction error (ME), defined as the difference between the postoperative and target SEQ, was calculated. Postoperative SEQ for GPT-5.2 was derived using a predefined conversion formula. Mean absolute error (MAE) and median absolute error (MedAE) were also calculated. In addition, a subgroup analysis was conducted according to axial length (AL: 22–23.9 mm, 24.0–25.9 mm, and ≥26.0 mm). Eyes with AL < 22 mm were excluded due to insufficient sample size (*n* = 5).

A GEE linear regression model was used to compare prediction errors (ME, MAE, and MedAE) between groups and assess correlations with AL, Km, ACD, and LT. To evaluate the proportion of eyes with postoperative SEQ within ±0.25 D, ±0.50 D, and ±1.0 D, a GEE linear regression model with a binomial distribution was used.

## 3. Results

A total of 293 eyes from 174 patients were included, with a mean age of 72 ± 8 years (35 to 90 years) (Table 1). The cohort comprised 77 males (47%) and 97 females (53%). The mean implanted IOL power was 19.64 ± 4.07 D (5.0 to 34.0 D). The toric subgroup included 51 eyes from 37 patients, with a mean implanted cylinder power of 2.56 ± 1.21 D (1.5 to 6.0 D).

### 3.1. Refractive and IOL Power Outcomes

The mean implanted IOL power selected by GPT-5.2 was 19.24 ± 4.01 D (5.0 to 30.5 D), which differed significantly from the surgeon’s selected IOL power of 19.64 ± 4.07 D (5.0 to 34.0 D) (*p* < 0.001) (Table 2).

Postoperative SEQ was −0.33 ± 0.59 D, which was more myopic than GPT-5.2 predicted SEQ (−0.05 ± 0.57 D, *p* = 0.009) (Table 2, Figure 1). Surgeon-selected IOLs had a higher proportion of eyes achieving postoperative SEQ within ±0.25 D (51.9% vs. 44.7%; odds ratio [OR] 0.75; *p* < 0.01) and ±1.0 D (97.3% vs. 92.2%; OR, 0.32; *p* < 0.01) than GPT-5.2.

### 3.2. Refractive Agreement Between Surgeon and GPT-5.2 Selected IOL Power

The ME between postoperative SEQ and target SEQ was −0.15 ± 0.03 D (mean ± SE), indicating a more myopic outcome than GPT-5.2 (0.12 ± 0.04 D, *p* < 0.001) (Table 2). GPT-5.2 demonstrated significantly higher MAE (0.45 ± 0.03 D vs. 0.35 ± 0.02 D, *p* = 0.002) and MedAE (0.35 D vs. 0.25 D) than for surgeon-selected IOLs. Because simulated postoperative SEQ for GPT-5.2 was derived from the measured postoperative SEQ using a fixed conversion factor, the between-group difference in ME (0.27 D) is an arithmetic restatement of the mean difference in selected IOL power rather than an independent outcome. Selections were identical in 158 of 293 eyes (54%), so between-method comparisons are driven by the 135 eyes in which selections differed.

Subanalysis by axial length showed lower MAE for the surgeon than for GPT-5.2 in eyes with AL 24.0–25.9 mm (*p* < 0.001) but was not statistically different for AL 22–23.9 mm (*p* = 0.100) and ≥26.0 mm (*p* = 0.137) (Figure 2). No significant correlations were observed between groups for Km, ACD, and LT as predictors (all *p* > 0.05).

### 3.3. Toric Cylinder Power and Axis Placement Selection

Among 51 toric eyes, mean cylinder power did not differ significantly between the surgeon and GPT-5.2 (2.56 ± 1.21 D vs. 2.35 ± 1.26 D, *p* = 0.13) (Table 2). Cylinder magnitude was identical in 51% of eyes and differed by −0.75 D in 25.5% of eyes (Supplemental File S3A). The absolute differences in the placement axis were <5° in 62% of eyes, 5° to <10° in 23%, 10° to <20° in 12%, and 20° to <35° in 2% (Supplemental File S3B).

Postoperative astigmatism centroids were 0.07 D at 70° ± 0.75 D for the surgeon compared to 0.05 D at 62° ± 0.96 D for GPT-5.2 (Figure 3). Mean absolute residual astigmatism was significantly higher for GPT-5.2 (0.78 ± 0.55 D) than for the surgeon (0.58 ± 0.47 D) (*p* < 0.001).

Axis flip was observed more frequently with GPT-5.2 (47.1%, 24/51) than with the surgeon (39.2%, 20/51) (Figure 4). Axis flip from preoperative WTR occurred in 39.1% (9/23) of eyes in both groups. In eyes with preoperative ATR, axis flip occurred in 53.8% (7/13) of GPT-5.2 cases compared with 38.5% (5/13) of surgeon cases, and in oblique eyes in (8/15, 53.3%) versus (6/15, 40.0%). Astigmatism subtype (WTR:ATR:oblique) was preserved in 52.9% of GPT-5.2 cases (27/51; 14:6:7) and 60.8% of surgeon cases (31/51; 14:8:9).

To quantify repeatability, we re-queried a random subsample of 100 eyes three times using the same snapshot and prompt. GPT-5.2 returned an identical IOL power in 96% of eyes and a power within ±0.5 D in 100% of eyes. Among the 16 toric eyes in the subsample, cylinder power and axis was within 5° in 100% of eyes.

## 4. Discussion

This study evaluated whether a general-purpose LLM could replicate surgeon decision-making in IOL power selection when given identical preoperative data. Rather than testing GPT-5.2 as a computational tool, we instructed it to function as a clinical decision-maker and produce a final IOL recommendation. On average, GPT-5.2 selected 0.40 D less IOL power than the surgeon. Because we derived simulated refractive outcomes from measured outcomes using a fixed conversion, this power difference and the hyperopic shift in ME express one observation. The direction of the selection carries the clinical meaning: cataract surgeons commonly target mild myopia to reduce the risk of postoperative hyperopia [4] and to meet patient expectations, whereas GPT-5.2 favored systematically lower powers. The conversion does not determine MAE or the proportion of eyes within ±0.25 D, and both favored the surgeon, with MAE 0.10 D higher for GPT-5.2. Its selections therefore fell further from target rather than simply landing on the hyperopic side of it, suggesting that the surgeon’s choices incorporated patient-specific judgment the model did not consistently reproduce.

Although refractive predictability favored surgeon-selected IOLs at the ±0.25 D and ±1.00 D thresholds, at least 90% of eyes in both groups achieved outcomes within ±1.00 D. Given that ±0.50 D represents the widely accepted benchmark for refractive accuracy [7], comparable performance at this threshold suggests that GPT-5.2 approaches clinically acceptable accuracy in routine cases.

In astigmatic eyes, GPT-5.2 resulted in approximately 0.2 D greater residual astigmatism and greater dispersion on vector analysis. Centroid orientation was directionally comparable between groups, indicating that the LLM can reasonably identify the astigmatic axis. In addition, GPT-5.2 preserved axis orientation in a proportion similar to that of the surgeon, but showed a higher proportion of axis flips in preoperative ATR eyes (53.8% vs. 38.5%) and oblique eyes. In some cases, conversion from ATR to WTR may be visually advantageous [20]. However, the higher frequency of axis flips for GPT-5.2 reflects a less conservative approach to selecting cylinder power. In contrast, the surgeon tended to favor lower cylinder magnitudes, likely to minimize axis flip [4], reflecting risk-aware clinical judgment that GPT-5.2 appears not to consistently reproduce.

In a subset of eyes in which GPT-5.2 was also prompted to provide a rationale, GPT-5.2 showed a tendency to anchor disproportionately on individual IOL formula outputs and defaulted to choosing the lens power that most closely matched a single formula row, even when additional contextual data were available. This was a qualitative observation and was not quantified in the primary analysis. In contrast, the surgeon considered all available formula outputs, placing more emphasis on those in agreement and those best validated for the eye’s biometric profile. Reliance on a single formula output may underutilize the complementary data that help clinicians evaluate reliability and reconcile discrepancies between formulas. As a result, the recommendation made by GPT-5.2 may appear numerically justified yet be less robust in eyes with borderline measurements or discordant outputs. The difference in reasoning between surgeon and GPT-5.2 highlights the need for cross-formula comparison and measurement-quality assessment, processes that a general-purpose LLM may not consistently perform, rather than relying on any single output as sufficient evidence for the final IOL selection.

Prior ophthalmology studies have evaluated the clinical reasoning tasks of LLMs, predominantly focusing on image-based diagnostics and task-specific predictive learning formulas [9,11,21,22]. The present study differs in that it evaluates whether a general-purpose LLM could assume the integrative role of the surgeon and generate an accurate, clinically grounded final IOL recommendation. While LLMs can interpret clinical vignettes and answer related questions, they often lack sufficient information for patient-specific decision-making [23]. GPT-5.2 was developed as a broad-domain LLM that can cover a wide range of knowledge, and while helpful to the general public, it is not optimized for ophthalmic surgical planning. It was nonetheless selected because it represented the most current flagship general-purpose LLM available at the time of the study and its API supported the automated, independent, multimodal queries required by our design. From a clinical perspective, GPT-5.2 does not currently achieve surgeon-level refractive precision, particularly in toric magnitude. Nevertheless, it demonstrated measurable capacity for this task, and its performance profile reflects the inherent limitations of a model not specifically trained in this domain. Our results raise the possibility of a domain-specific LLM that is fine-tuned for IOL selection. The success of data-driven AI formulas such as Hill-RBF demonstrates that large-scale postoperative outcomes can be leveraged effectively for prediction [22]. Future work could focus on designing a system that integrates outputs from clinically validated formulas with relevant patient context, including ocular history, prior refractive surgery, corneal measurements, and surgeon preference, to help choose an individualized IOL strategy that supplements clinical judgment.

Three prompt characteristics were particularly influential in this study. First, explicit instruction to review all supplied inputs was required to prevent the model from defaulting to a single formula row. Initial iterations of less constrained prompts produced selections that closely mirrored one formula output despite the availability of multiple formulas and device measurements. Second, the LLM’s outputs had to be explicitly bounded to options that physically exist. The model was instructed to select only from the manufacturer’s listed toric cylinder powers and not to extrapolate or generate unavailable values, and to restrict biometric inputs to IOLMaster or Lenstar measurements rather than substituting values from topography devices. Without such constraints, an LLM may produce a numerically plausible recommendation that does not exist commercially or mix measurements across instruments with different keratometric conventions. Third, the required output had to be constrained to a discrete recommendation—spherical power, cylinder power, and axis—rather than an open-ended response, which otherwise yielded qualified discussion without a final selection. Conversely, over-specification, such as directing the model toward a particular formula or decision rule, would have reduced the task to arithmetic rather than clinical reasoning. Prompt design should therefore be considered a primary variable in LLM clinical evaluation.

The use of an API in our study, rather than the standard web interface, was intentional and has practical implications clinically. In addition to its efficiency and accuracy in data manipulation when compared to manual entry [24], an API-based workflow would be well-suited for integration into electronic medical records software, where LLMs could assist by organizing relevant clinical data, stratifying formula outputs, and supporting final IOL power selection. With this framework, refractive prediction would remain driven by established methods, while the LLM would improve the workflow. Whether this type of integrated approach improves outcomes or efficiency requires further investigation.

Several limitations warrant consideration. The retrospective design and single-surgeon, single-center cohort limit the generalizability of these findings, though the inclusion of a single surgeon provides a consistent and internally valid benchmark for comparison. Even so, validation against multiple surgeons is required to determine whether these findings generalize beyond a single surgeon’s practice. The toric subgroup was small, which may also limit statistical power. The absence of a significant difference in cylinder magnitude between GPT-5.2 and the surgeon (*p* = 0.13) should therefore be interpreted as inconclusive rather than as evidence of no difference. No adjustment for multiple comparisons was applied, and given the number of refractive, subgroup, and astigmatic endpoints examined, the potential for type I error is increased. However, the principal comparisons of IOL power, mean absolute error, and residual astigmatism remained significant at *p* ≤ 0.002 and are unlikely to be explained by multiplicity alone. Axis flip was reported descriptively as observed proportions without significance testing. The use of a 0.7 D spectacle-plane conversion per 1.0 D difference in IOL power may introduce error but enabled standardized comparison across groups. Given that 90% of eyes differed by ≤1.0 D in selected IOL power, the conversion range of 0.6–0.8 D would result in a maximum variation of ±0.1 D in estimated postoperative SEQ in most eyes, a magnitude unlikely to be clinically meaningful for individual eyes. However, small changes could affect categorical accuracy outcomes for eyes located near the ±0.25 D and ±0.50 D thresholds. Among the 5% of eyes with IOL power discrepancies exceeding 2.0 D between GPT-5.2 and the surgeon, the fixed conversion factor may have introduced greater estimation error. As this minority of cases were not systematically biased in a single direction, their influence on aggregate refractive outcome estimates is expected to be minimal. The use of API facilitated automation and analysis of larger datasets, but limited qualitative exploration of GPT-5.2’s reasoning. We did not formally prompt GPT-5.2 to provide an explanation for all IOL selections. Furthermore, LLMs function as “black box” systems, and their internal computational processes are not directly observable [25]. Although GPT-5.2 can generate explanations for its recommendations, these explanations are themselves model-selected outputs and do not provide direct access to the mechanisms underlying the decision. For these reasons, our understanding of GPT-5.2’s internal reasoning remains limited. The temperature was set to 0, which minimizes but does not eliminate output variability, as residual non-determinism is inherent to large language models and represents the practical ceiling of reproducibility currently achievable in any commercial LLM-based study [13].

In conclusion, GPT-as5.2 demonstrated the ability to synthesize complex refractive inputs and generate IOL recommendations that were still less accurate than an experienced surgeon. Although GPT-5.2 does not yet match the precision of surgeon-level performance, it shows growing capacity to approximate the clinical decision-making process in routine cataract surgery. General-purpose LLMs are not a substitute for surgical judgment but may serve as an adjunct tool. Domain-specific refinement and integration with modern IOL calculation tools may be required before such LLMs can be reliably implemented into practice.

## Figures and Tables

**Figure 1:**
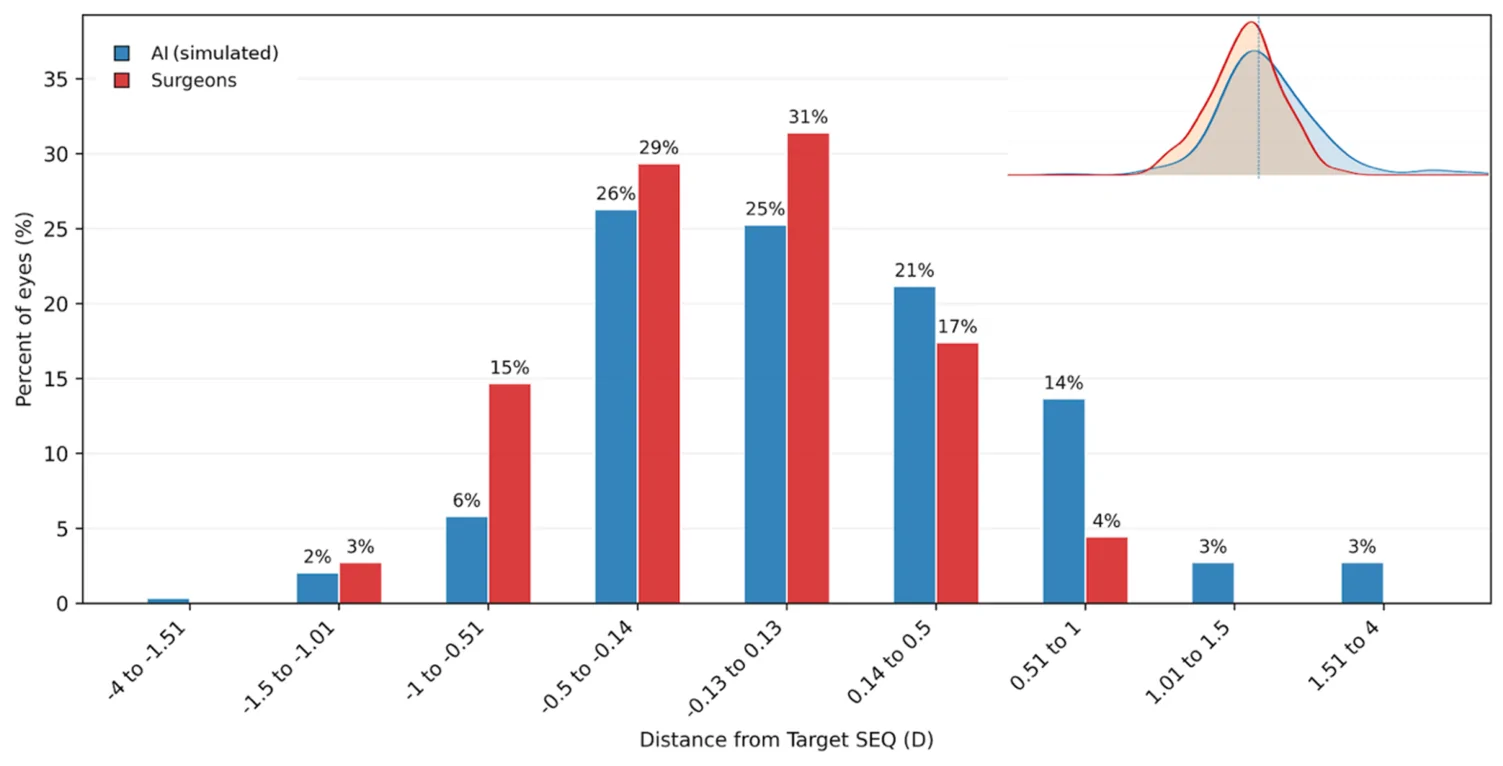
Refractive agreement between the surgeon and GPT-5.2 selected IOL power.

**Figure 2:**
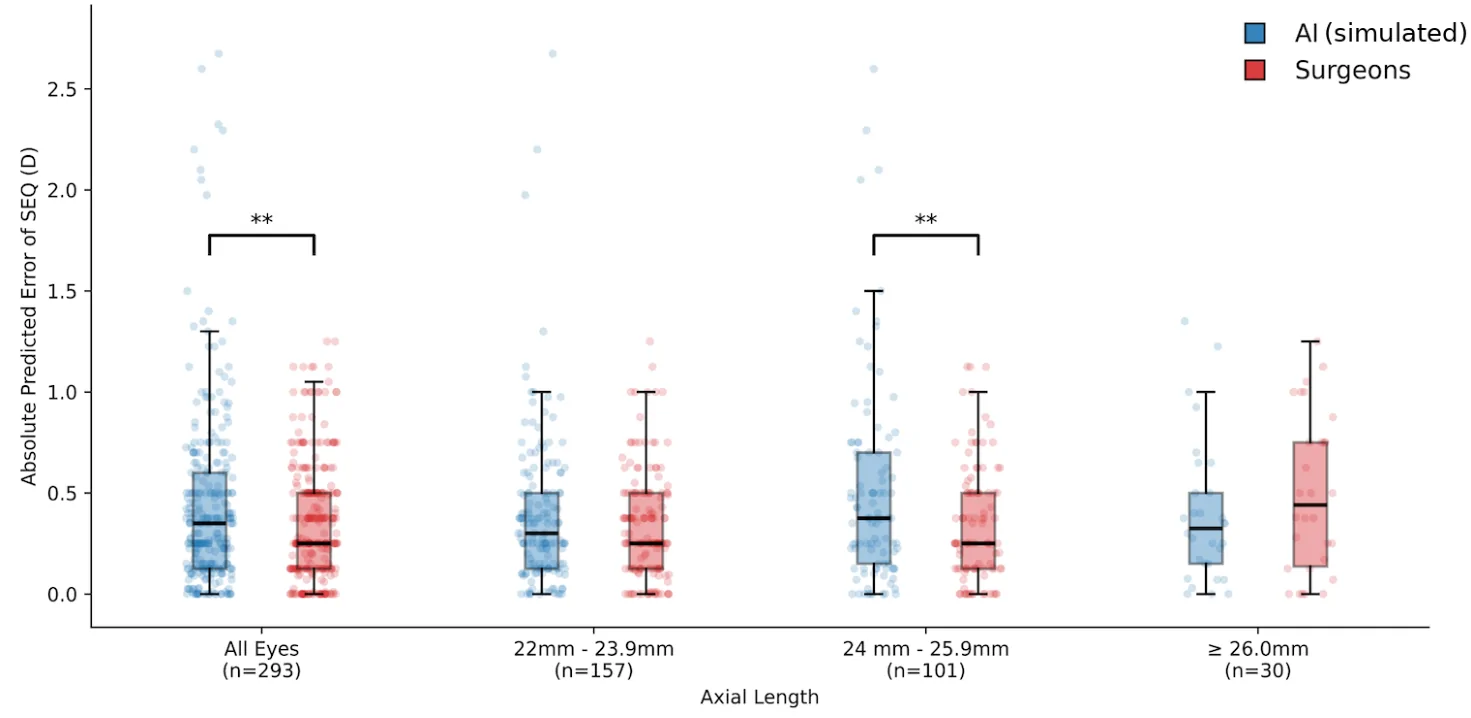
Absolute prediction error stratified by axial length (22–23.9 mm, 24.0–25.9 mm, and ≥26.0 mm) between the surgeon and GPT-5.2 selected IOL power. Boxes indicate the interquartile range with median lines, whiskers denote the range, and points represent individual eyes. All-eyes analysis included 293 eyes; AL subgroups included 288, excluding 5 eyes with AL < 22 mm. ** Indicates statistically significant differences between groups.

**Figure 3:**
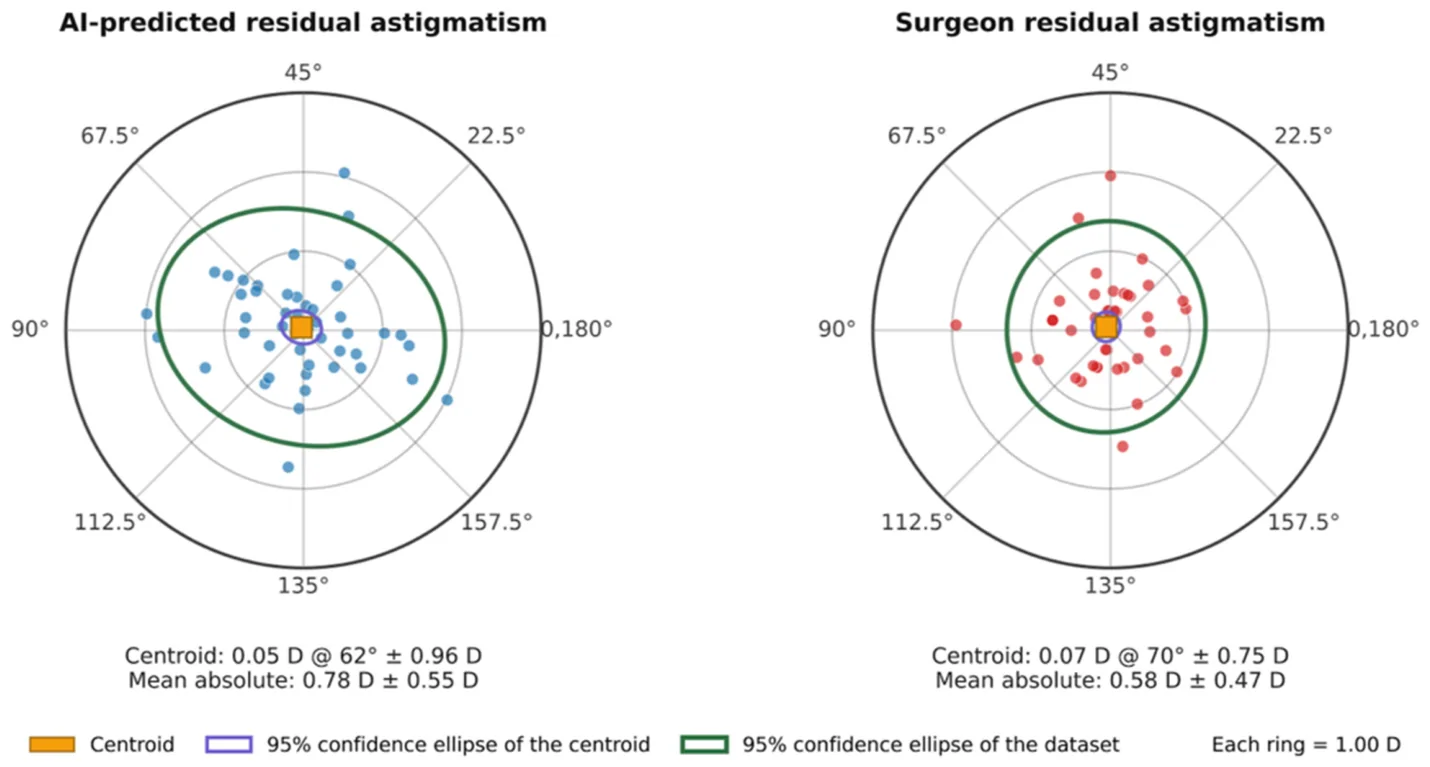
Double-angle vector plots between GPT-5.2 and the surgeon for eyes receiving toric IOLs.

**Figure 4:**
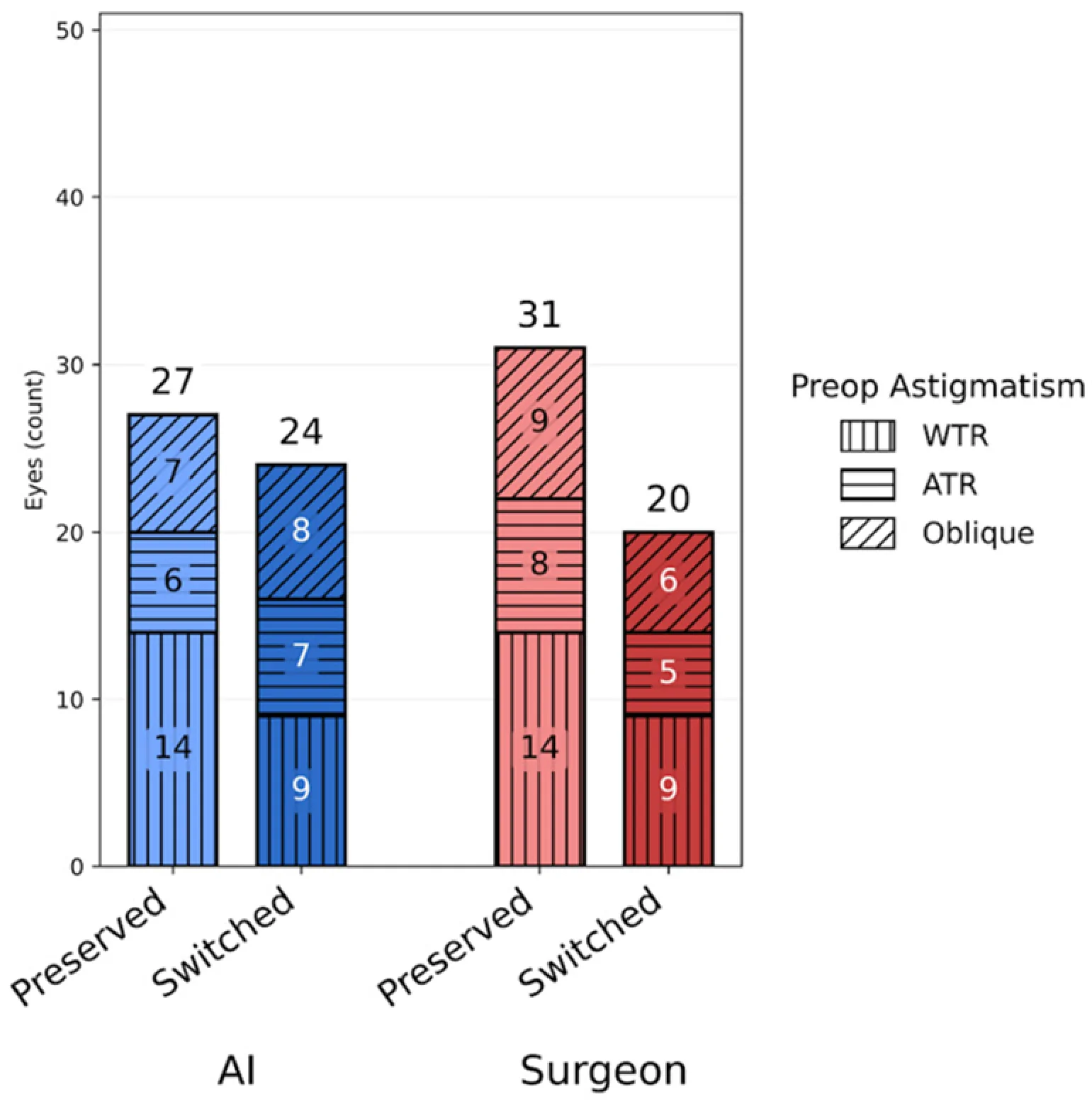
Distribution of axis preservation and axis flip between surgeon and GPT-5.2, stratified by preoperative with-the-rule (WTR), against-the-rule (ATR), and oblique astigmatism.

**Table 1:** Preoperative patient characteristics.

Parameter	Values
Total Patients/Eyes	174/293 ^a^
OD/OS, *n* (%)	144 (49)/149 (51)
Age (y)	72 ± 8 (35, 90)
Male/Female, *n* (%)	77 (47)/97 (53)
Myopia with and without astigmatism, *n* (%)	144 (49)/12 (4)
Hyperopic with and without astigmatism, *n* (%)	82 (28)/9 (3)
Mixed astigmatism, *n* (%)	37 (13)
Toric/Monofocal, *n* (%)	51 (17)/242 (83)
AL (mm)	24.08 ± 1.37 (21.57, 28.39)
ACD (mm)	3.25 ± 0.34 (2.18, 4.37)
LT (mm)	4.56 ± 0.49 (2.00, 5.92)
WTW (mm)	12.02 ± 0.42 (9.92, 13.30)
K1 (D)	43.55 ± 1.81 (33.73, 48.08)
K1 axis (°)	88.22 ± 54.08 (0, 180)
K2 (D)	44.6 ± 1.86 (35.98, 50.24)
K2 axis (°)	86.27 ± 47.23 (1, 180)
IOL power (D)	19.64 ± 4.07 (5.0, 34.0)
IOL Cylinder (D)	2.56 ± 1.21 (1.5, 6.0)

OD = right eye; OS = left eye; IOL = intraocular lens; D = diopters; AL = axial length; ACD = anterior chamber depth; LT = lens thickness; WTW = white to white; K1 = flat keratometry; K2 = steep keratometry. Values expressed as mean ± standard deviation (range). ^a^ 9 (3%) eyes were not refracted due to the severity of cataract.

**Table 2:** Comparison of refractive outcomes between surgeon-selected (measured) and GPT-5.2-selected (simulated) IOL power.

Parameter	AI (Simulated) (*N* = 293)	Surgeon (*N* = 293)	Odds Ratio	*p* Value
% of eyes within target SEQ				
Within ±0.25 D	44.7	51.9	0.75	<0.01
Within ±0.5 D	72.7	78.2	0.74	0.06
Within ±1.0 D	92.2	97.3	0.32	<0.01
Prediction error (D)				
ME ± robust SE (95% CI)	0.12 ± 0.04 (0.04, 0.21)	−0.15 ± 0.03 (−0.21, −0.10)	_—_	<0.001
MAE ± robust SE (95% CI)	0.45 ± 0.03 (0.39, 0.51)	0.35 ± 0.02 (0.32, 0.39)	_—_	0.002
MedAE (P_25_, P_75_)	0.35 (0.125, 0.6)	0.25 (0.125, 0.5)	_—_	_—_
Postop SEQ ± SD, range (D)	−0.05 ± 0.57 (−2.33, 2.83)	−0.33 ± 0.59 (−3.00, 1.00)	_—_	0.009
IOL power ± SD, range (D)	19.24 ± 4.01 (5.0, 30.5)	19.64 ± 4.07 (5.0, 34.0)	_—_	<0.001
IOL Cylinder ± SD, range (D)	2.35 ± 1.26 (1.5, 6.0)	2.56 ± 1.21 (1.5, 6.0)	_—_	0.13

Abbreviations: AI = Artificial Intelligence; IOL = intraocular lens; D = diopters; OR = odds ratio; CI = confidence interval; SEQ = spherical equivalent; ME = mean predictive error; MAE = mean absolute error; MedAE = median absolute error.

## Data Availability

The original contributions presented in this study are included in the article/Supplementary Material. Further inquiries can be directed to the corresponding author.

## Supplementary Materials

The following supporting information can be downloaded at https://www.mdpi.com/article/10.3390/jcm15197463/s1, Supplemental File S1: The structured prompt template included placeholders for patient demographics, preoperative manifest refraction, and planned IOL parameters for all eyes. Supplemental File S2: A representative example of the preoperative imaging and biometry used for surgical planning and provided to GPT-5.2 is shown. When available, measurements from all devices were reviewed by the surgeon and therefore provided to GPT-5.2. Optical biometry was performed using (A) the Lenstar LS 900 (Haag-Streit) and/or (B) the IOL Master 700 (Carl Zeiss Meditec AG). Corneal topography was obtained using (C) OPD-Scan III (Nidek Co., Ltd.), (D) Galilei G4 (Ziemer USA, Inc.), or (E) Pentacam HR (Oculus Surgical, Inc.). Three Pentacam displays were included: the 4 Maps Refractive, Refractive, and Belin/Ambrósio Enhanced Ectasia Display. Supplemental File S3: Differences in toric IOL cylinder magnitude (A) and axis selection (B) between GPT-5.2 and the surgeon. Positive values indicate a higher axis or cylinder magnitude selected by GPT-5.2 relative to the surgeon. Supplemental File S4: Completed TRIPOD-LLM reporting checklist.
