# Supplementary material for: Retrospective Assessment of LLM Decision-Making for IOL Power Selection in Cataract Surgery in Comparison with Surgeon Performance
Source: J Clin Med. 2026 Sep 25;15(19):7463. doi: 10.3390/jcm15197463 (PMC13666018; doi:10.3390/jcm15197463)
Supplement: Supplementary file 1 [file jcm-15-07463-s001.zip › Supplemental File S2.pdf]

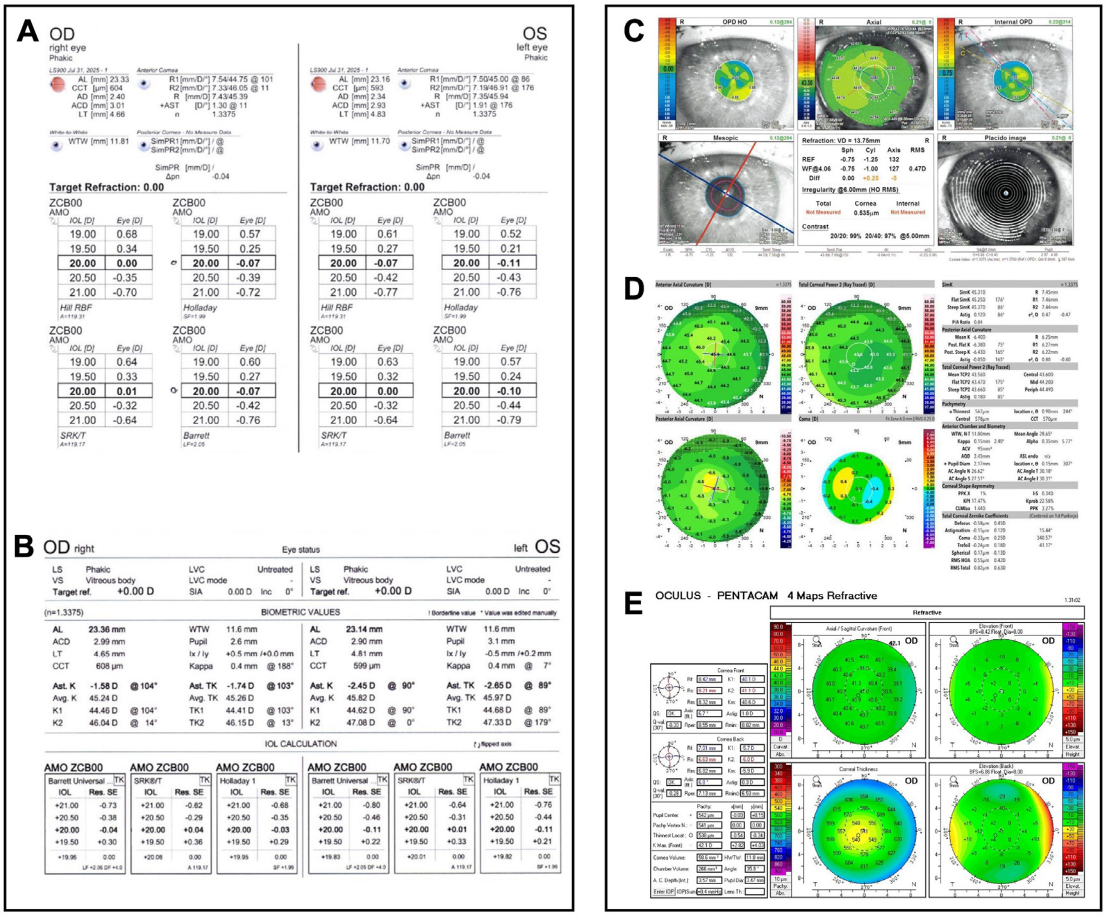

Supplemental File S2. A representative example of the preoperative imaging and biometry used for surgical planning and provided to GPT-5.2 is shown. When available, measurements from all devices were reviewed by the surgeon and therefore provided to GPT-5.2. Optical biometry was performed using (A) the Lenstar LS 900 (Haag-Streit) and/or (B) the IOL Master 700 (Carl Zeiss Meditec AG). Corneal topography was obtained using (C) OPD-Scan III (Nidek Co., Ltd.), (D) Galilei G4 (Ziemer USA, Inc.), or (E) Pentacam HR (Oculus Surgical, Inc.). Three Pentacam displays were included: the 4 Maps Refractive, Refractive, and Belin/Ambrósio Enhanced Ectasia Display
