# Supplementary material for: Retrospective Assessment of LLM Decision-Making for IOL Power Selection in Cataract Surgery in Comparison with Surgeon Performance
Source: J Clin Med. 2026 Sep 25;15(19):7463. doi: 10.3390/jcm15197463 (PMC13666018; doi:10.3390/jcm15197463)
Supplement: Supplementary file 1 [file jcm-15-07463-s001.zip › Supplemental File S3.pdf]

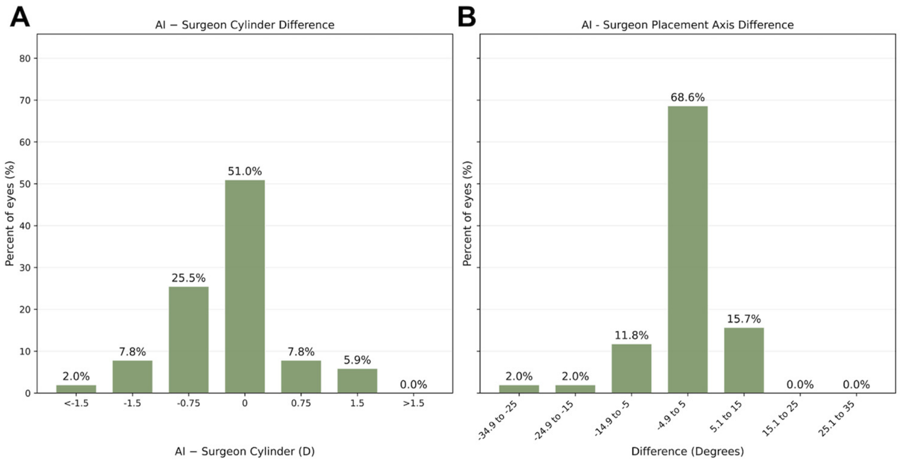

Supplemental File S3. Differences in toric IOL cylinder magnitude (A) and axis selection (B) between GPT-5.2 and the surgeon. Positive values indicate a higher axis or cylinder magnitude selected by GPT-5.2 relative to the surgeon.
