# Supplementary material for: Retrospective Assessment of LLM Decision-Making for IOL Power Selection in Cataract Surgery in Comparison with Surgeon Performance
Source: J Clin Med. 2026 Sep 25;15(19):7463. doi: 10.3390/jcm15197463 (PMC13666018; doi:10.3390/jcm15197463)
Supplement: Supplementary file 1 [file jcm-15-07463-s001.zip › Supplemental File S4.pdf]

Supplementary File S4. TRIPOD+LLM Checklist

Section / Topic	Item Number	Checklist Item	Reported on Page
**Abstract**			
Title	2a	Identify the study as developing, fine-tuning, and/or evaluating the performance of an LLM, specifying the task, the target population, and the outcome to be predicted.	Title page
Abstract	2b	Provide a brief explanation of the healthcare context, use case and rationale for developing or evaluating the performance of an LLM.	1
Objectives	2c	Specify the study objectives, including whether the study describes LLMs development, tuning, and/or evaluation.	1
Methods	2d	Describe the key elements of the study setting.	1
Methods	2e	Detail all data used in the study, specify data splits and any selective use of data.	1
Methods	2f	Specify the name and version of LLM used.	1
Methods	2g	Briefly summarize the LLM-building steps, including any fine-tuning, reward modeling, reinforcement learning with human feedback (RLHF), etc.	Not Required
Methods	2h	Describe the specific tasks performed by the LLMs (e.g., medical QA, summarization, extraction), highlighting key inputs and outputs used in the final LLM.	1
Methods	2i	Specify the evaluation datasets/populations used, including the endpoint evaluated, and detail whether this information was held out during training/tuning where relevant, and what measure(s) were used to evaluate LLM performance.	1
Results	2j	Give an overall report and interpretation of the main results.	1
Discussion	2k	Explicitly state any broader implications or concerns that have arisen in light of these results.	1
Other	2l	Give the registration number and name of the registry or repository (if relevant).	Not Required
**Introduction**			
Background	3a	Explain the healthcare context / use case (e.g., administrative, diagnostic, therapeutic, clinical workflow) and rationale for developing or evaluating the LLM, including references to existing approaches and models.	2
Background	3b	Describe the target population and the intended use of the LLM in the context of the care pathway, including its intended users in current gold standard practices (e.g., healthcare professionals, patients, public, or administrators).	2, 3
Objectives	4	Specify the study objectives, including whether the study describes the initial development, fine-tuning, or validation of an LLM (or multiple stages).	3
**Methods**			
Data	5a	Describe the sources of data separately for the training, tuning, and/or evaluation datasets and the rationale for using these data (e.g., web corpora, clinical research/trial data, EHR data).	3, 4, 5
Data	5b	Describe the relevant data points and provide a quantitative and qualitative description of their distribution and other relevant descriptors of the dataset (e.g., source, languages, countries of origin).	4, 8, Table 1
Data	5c	Specifically state the date of the oldest and newest item of text used in the development process (training, fine-tuning, reward modeling) and in the evaluation datasets.	3
Data	5d	Describe any data pre-processing and quality checking, including whether this was similar across text corpora, institutions, and relevant sociodemographic groups.	5
Data	5e	Describe how missing and imbalanced data were handled and provide reasons for omitting any data.	3
Analytical Methods	6a	Report the LLM name, version, and last date of training or use during inference.	4, 5
Analytical Methods	6b	Specify the type of LLM architecture, and LLM building steps, including any hyperparameter tuning (e.g., temperature, length limits, penalties), prompt engineering, and any inference settings (e.g., seed, temperature, max token length) as relevant.	4, 5
Analytical Methods	6c	Report details of LLM development process from text input to outcome generation, such as training, fine-tuning procedures, and alignment strategy (e.g., reinforcement learning, direct preference optimization, etc.) and alignment goals (e.g., helpfulness, honesty, harmlessness, etc.).	Not Required
Analytical Methods	6d	Specify the initial and post-processed output of the LLM (e.g., probabilities, classification, unstructured text).	5, 6
Analytical Methods	6e	Provide details and rationale for any classification and how the probabilities were determined and thresholds identified.	Not Required
Analytical Methods	6f	Include metrics that capture the quality of generative outputs, such as consistency, relevance, and accuracy, compared to gold standards.	7, 8, 9
Analytical Methods	6g	Report the outcome metrics' relevance to downstream task at deployment time	7, 12, 13

		and correlation of metric to human evaluation of the text for the intended use.	
LLM Output	7a	Clearly define the outcome, how the LLM predictions were calculated (e.g., formula, code, object, API), and evaluation metrics.	5, 6, 7
LLM Output	7b	If outcome assessment requires subjective interpretation, describe the qualifications of the assessors, any instructions provided, relevant information on demographics of the assessors, and inter-assessor agreement.	3, 4
LLM Output	7c	Specify how performance was compared to other LLMs, humans, and other benchmarks or standards.	7, 8, 9, 10
Annotation	8a	If annotation was done, report how text was labeled, including providing specific annotation guidelines with examples.	N/A
Annotation	8b	If annotation was done, report how many annotators labeled the dataset(s), including the proportion of data in each dataset that were annotated by more than 1 annotator.	N/A
Annotation	8c	If annotation was done, provide information on the background and experience of the annotators, and the inter-annotator agreement.	N/A
Prompting	9a	If research involved prompting LLMs, provide details on the processes used during prompt design, curation, and selection.	4, 5, Supplementary File 1
Prompting	9b	If research involved prompting LLMs, report what data were used to develop the prompts.	4, 5, Supplementary File 1
Summarization	10	Describe any preprocessing of the data before summarization.	Not Required
Instruction Tuning / Alignment	11	If instruction tuning/alignment strategies were used, what were the instructions and interface used for evaluation, and what were the characteristics of the populations doing evaluation?	Not Required
Compute	12	Report compute, or proxies thereof (e.g., time on what and how many machines, cost on what and how many machines, inference time, floating-point operations per second (FLOPs)), required to carry out methods.	Not reported
Ethics Approval	13	Name the institutional research board or ethics committee that approved the study and describe the participant-informed consent or the ethics committee waiver of informed consent.	3
Open Science	14a	Give the source of funding and the role of the funders for the present study.	N/A
Open Science	14b	Declare any conflicts of interest and financial disclosures for all authors.	16
Open Science	14c	Indicate where the study protocol can be accessed or state that a protocol was not prepared.	Not Required
Open Science	14d	Provide registration information for the study, including register name and registration number, or state that the study was not registered.	Not Required
Open Science	14e	Provide details of the availability of the study data.	N/A
Open Science	14f	Provide details of the availability of the code to reproduce the study results.	Supplementary File 1
Public Involvement	15	Provide details of any patient and public involvement during the design, conduct, reporting, interpretation, or dissemination of the study or state no involvement.	Not Required
**Results**			
Participants	16a	When using patient/EHR data, describe the flow of text/EHR/patient data through the study, including the number of documents/questions/participants with and without the outcome/label and follow-up time.	3, 8
Participants	16b	When using patient/EHR data, report the characteristics overall and, for each data source or setting, and for development/evaluation splits, including the key dates, key predictors, and sample size.	8, Table 1
Participants	16c	For LLM evaluation, show a comparison of the distribution of important predictors between development and evaluation data.	N/A
Participants	16d	When using patient/EHR data, specify the number of participants and outcome events in each analysis (e.g., for LLM development, hyperparameter tuning, LLM evaluation).	8, 9, Table 2
Performance	17	Report LLM performance according to pre-specified metrics (see item 7a) and/or human evaluation (see item 7d).	8, 9, 10, Tables 1-2, Figures 1-4
LLM Updating	18	If applicable, report the results from any LLM updating, including the updated LLM and subsequent performance.	N/A
**Discussion**			
Interpretation	19a	Give an overall interpretation of the main results, including issues of fairness in the context of the objectives and previous studies.	10, 11, 12
Limitations	19b	Discuss any limitations of the study and their effects on any biases, statistical uncertainty, and generalizability.	13, 14
Usability of the LLM in context	19c	Describe any known challenges in using data for the specified task and domain context with reference to representation, missingness, harmonization, and bias.	12, 13, 14
Usability of the LLM in context	19d	Define the intended use for the implementation under evaluation, including the intended input, end-user, level of autonomy/human oversight.	13, 14, 15

Usability of the LLM in context	19e	If applicable, describe how poor quality or unavailable input data should be assessed and handled when implementing the LLM, i.e., what is the usability of the LLM in the context of current clinical care.	12
Usability of the LLM in context	19f	If applicable, specify whether users will be required to interact in the handling of the input data or use of the LLM, and what level of expertise is required of users.	13, 14
Usability of the LLM in context	19g	Discuss any next steps for future research, with a specific view to applicability and generalizability of the LLM.	12, 13, 14
